# Pro-angiogenic therapeutics for preeclampsia

**DOI:** 10.1186/s13293-018-0195-5

**Published:** 2018-08-25

**Authors:** Adrian C. Eddy, Gene L. Bidwell, Eric M. George

**Affiliations:** 1 Department of Physiology and Biophysics, 2500 N State St, Jackson, MS 39216 USA; 2 Department of Cell and Molecular Biology, 2500 N State St, Jackson, MS 39216 USA; 3 Department of Neurology, University of Mississippi Medical Center, 2500 N State St, Jackson, MS 39216 USA

**Keywords:** Preeclampsia, Angiogenic balance, sFlt-1, VEGF, PlGF, Therapeutics

## Abstract

Preeclampsia is a pregnancy-induced hypertensive disorder resulting from abnormal placentation, which causes factors such as sFlt-1 to be released into the maternal circulation. Though anti-hypertensive drugs and magnesium sulfate can be given in an effort to moderate symptoms, the syndrome is not well controlled. A hallmark characteristic of preeclampsia, especially early-onset preeclampsia, is angiogenic imbalance resulting from an inappropriately upregulated sFlt-1 acting as a decoy receptor binding vascular endothelial growth factor (VEGF) and placental growth factor (PlGF), reducing their bioavailability. Administration of sFlt-1 leads to a preeclamptic phenotype, and several models of preeclampsia also have elevated levels of plasma sFlt-1, demonstrating its role in driving the progression of this disease. Treatment with either VEGF or PlGF has been effective in attenuating hypertension and proteinuria in multiple models of preeclampsia. VEGF, however, may have overdose toxicity risks that have not been observed in PlGF treatment, suggesting that PlGF is a potentially safer therapeutic option. This review discusses angiogenic balance as it relates to preeclampsia and the studies which have been performed in order to alleviate the imbalance driving the maternal syndrome.

## Background

Preeclampsia is a hypertensive disorder of pregnancy, which impacts 2–8% of pregnancies worldwide, and typically presents after 20 weeks of gestation [1]. Though the disease formerly required both new onset of hypertension and proteinuria to be diagnosed, this is no longer true. Today, diagnosis requires a systolic blood pressure greater than or equal to 140 mmHg or diastolic pressure of at least 90 mmHg along with one or more of the following: proteinuria, thrombocytopenia, renal insufficiency, liver function impairment, pulmonary edema, or cerebral or visual disturbances. Severe preeclampsia is characterized by an earlier onset, presenting prior to 20 weeks of gestation, and systolic blood pressure greater than or equal to 160 mmHg. The onset of seizures marks the progression to eclampsia [2]. Whereas the rates of occurrence of preeclampsia overall have remained unchanged, the cases of severe preeclampsia have begun to rise over the last several decades [3].

**Fig. 1:**
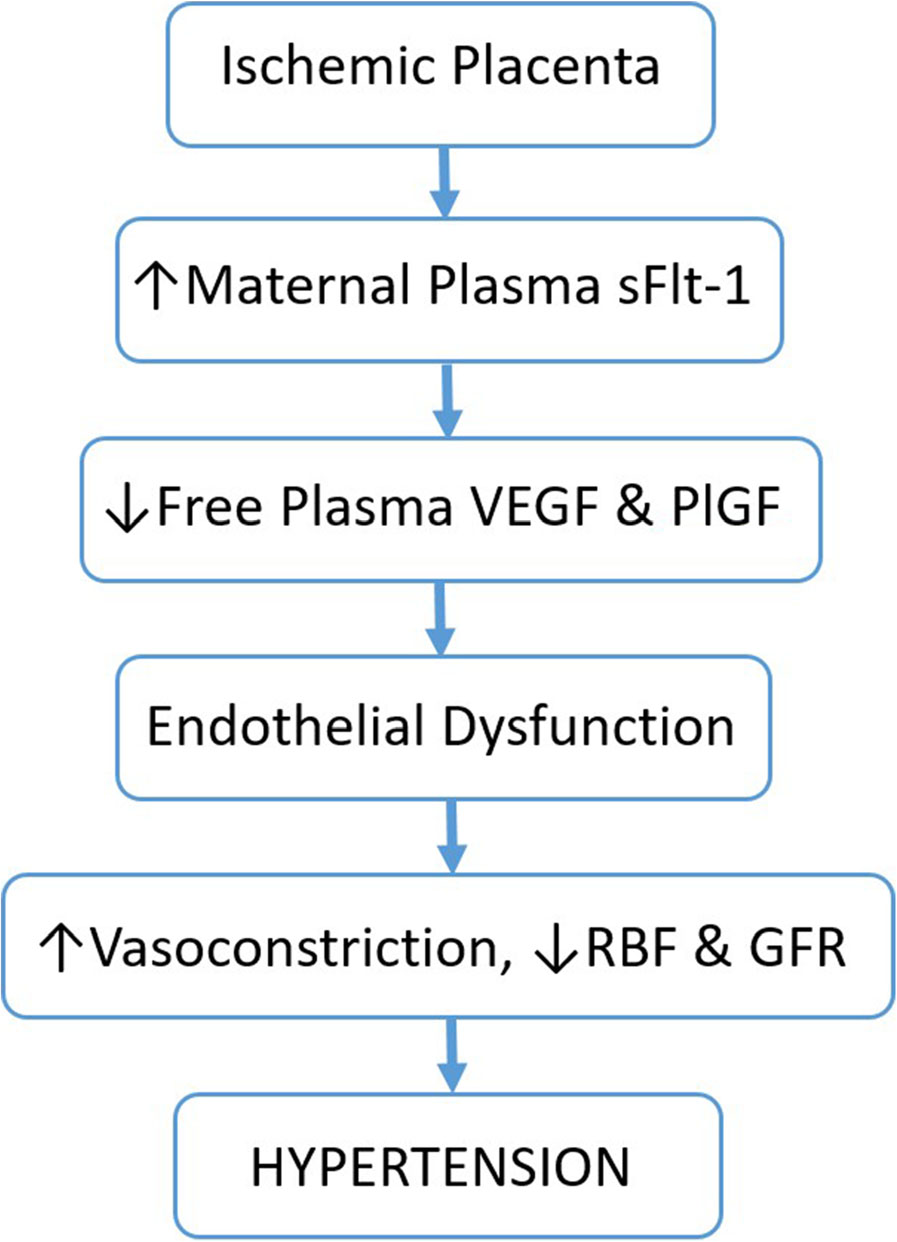
Contribution of sFlt-1 to the maternal syndrome of preeclampsia. In preeclampsia, it is believed that the maternal vasculature fails to remodel and provide adequate blood flow to the placenta, causing chronic ischemia. As a result, the placenta produces various factors, notably sFlt-1, which contribute to the maternal syndrome. sFlt-1 acts as a decoy receptor for VEGF and PlGF, causing decreased bioavailable VEGF protein. This leads to maternal vascular endothelial dysfunction, causing aberrant vasoconstriction, changes in renal function, and ultimately hypertension

Preeclampsia is thought to result from improper infiltration of trophoblast cells into the maternal decidua during placentation, causing impaired spiral artery remodeling and decreased blood flow to the developing fetal-placental unit [4, 5]. The under-perfused placenta then releases a number of factors into the maternal circulation, leading to maternal endothelial dysfunction and the associated symptoms of the disorder [4], as depicted in Fig. 1. Though the reason for the abnormal placentation is not well understood, and the underlying cause of the disorder is unclear, there are several known risk factors which are associated with an increased risk of developing preeclampsia. These include preexisting diabetes, obesity, African descent, and previous preeclamptic pregnancy [6, 7].

Currently, therapeutic options for the treatment of preeclampsia are limited. Though anti-hypertensive drugs and magnesium sulfate can be given to aid in prevention and treatment of seizures, the only ultimate therapy for preeclampsia is the delivery of the fetus and placenta [2]. Early delivery should be considered if there are signs of fetal compromise [8, 9]. Whenever possible, vaginal delivery is preferred to cesarean due to the physical stress that occurs during a cesarean section [9]. If the disease progresses to eclampsia, which is characterized by the development of seizures, magnesium sulfate has been shown to be an effective prophylactic treatment to reduce seizure risk and severity [10]. In addition to the complications during pregnancy, preeclampsia has lasting effects on both mother and fetus. Mothers with preeclamptic pregnancies have increased risk of cardiovascular disease, including chronic heart failure, hypertension, and stroke [11]. Babies born of preeclamptic women are often born with low birth weight, which is correlated with increase in stroke, heart disease, and metabolic disorders in early adulthood [12, 13]. Aside from the mortality risks associated with preeclampsia, the lifelong effects it has on both mother and fetus pose a large economic burden. Given these facts, it is clear that there is an immense need for the development of therapeutic targets for the treatment of preeclampsia.

## VEGF, its receptors, and their functions

The decreased blood flow to the placenta mentioned above leads to a local hypoxic environment in the placenta [14, 15]. In response to hypoxia, vascular endothelial growth factor (VEGF) is transcriptionally upregulated; this growth factor is a particularly effective pro-angiogenic factor and is important to maintain the health of existing vessels [16]. Though VEGF can bind three receptors, its actions are mediated through two tyrosine kinase receptors, VEGF receptor-1 (VEGFR-1) and VEGF receptor-2 (VEGFR-2). VEGFR-1 is also known as Flt-1 (fms-like tyrosine kinase 1), and VEGFR-2 is commonly referred to as Flk-1 (fetal liver kinase 1). VEGFR-1 also has a splice variant sVEGFR-1/sFlt-1, which contains only the extracellular domain of the receptor, making it soluble in plasma [17]. Because sFlt-1 contains the binding site for VEGF, it is still able to bind all isoforms of the growth factor, as well as its close relative placental growth factor (PlGF) [18].

Although VEGF is commonly referred to as a single factor, it is actually a family of proteins. This family consists of VEGF-A (often referred to as simply VEGF), VEGF-B, VEGF-C, and VEGF-D [19]. VEGF-C and VEGF-D are both involved in the growth of lymphatic vessels [19], and for that reason, will not be discussed further in this review. PlGF is also a member of the VEGF family [19]. Members of the VEGF family exist as dimers, typically homodimers, though it is possible for heterodimers to form (VEGF/PlGF for example) [20]. VEGF and its family members are prone to rapid degradation and thus have a relatively short plasma half-life of approximately 30 min [21]. Because endothelial cells are among those that secrete VEGF and it acts in a paracrine fashion, the roles of VEGF are often thought to be limited to endothelial proliferation, migration, and increased vascular permeability. Non-vascular actions of VEGF, such as recruitment of inflammatory cells, however, also appear to be of growing importance [22]. The actions exerted by VEGF family members are elicited through their receptors. VEGF-A is able to bind both Flt-1 and Flk-1. VEGF-B and PlGF bind only Flt-1 [23]. As mentioned above, Flt-1 has a soluble form, sFlt-1, which will also bind the same ligands as its full length variant [24]. The receptors also have varying affinities for VEGF binding. While Flt-1 has an order of magnitude higher affinity to bind VEGF, Flk-1 has ten times the kinase activity of Flt-1 when its ligand is bound [25].

As discussed above, VEGF binds multiple receptors. It was previously thought that all angiogenic processes came from the activation of Flk-1, due to the fact that Flk-1-null mice die in utero at approximately day 8.5 from failure of endothelial progenitor cells to migrate and expand [26–28]. Similarly, mice lacking the Flt-1 receptor die between embryonic days 8.5–9. Their death, however, is due to excess formation of endothelial cells and disorganized formation of vascular tubules [29]. Because deletion of the tyrosine kinase domain of the Flt-1 receptor does not impede angiogenesis, it was previously concluded that Flt-1 acts as a VEGF decoy receptor to regulate the availability of VEGF for Flk-1, which mediates angiogenesis [30, 31]. Later evidence showed, however, that Flt-1 also participates in angiogenesis during adulthood [32]. Activating Flt-1 leads to nitric oxide (NO) release and stimulates organization of endothelial cells into capillary-like tubes [33]. VEGF binding to Flt-1 activates PI3K, which signals Akt, thereby inducing NO release by endothelial nitric oxide synthase (eNOS) [34]. The importance of VEGF signaling, particularly via Flk-1 due to its higher magnitude of kinase activity, is seen in the development of preeclampsia, when sFlt-1 interference antagonizes VEGF.

### sFlt-1 structure and function

#### Normal angiogenic balance

In order to understand the angiogenic imbalance that occurs in preeclampsia, it is crucial to first understand the normal signaling and the factors involved in maintaining angiogenic balance. Angiogenesis is the growth of new vessels from preexisting microvasculature [35] and can be initiated in multiple ways including shear stress [36] and tissue hypoxia [37]. Members of the VEGF family and some of their receptors are involved in preserving angiogenic homeostasis. The VEGF receptors can form either homodimers or heterodimers when they bind their substrates [38], and different combinations can result in either a pro- or anti-angiogenic effect. For example, when PlGF binds to Flt-1, it is considered pro-angiogenic [39], because this interaction increases the bioavailability of VEGF to bind to the more pro-angiogenic Flk-1. VEGF binding to Flt-1, on the other hand, is considered mildly anti-angiogenic [39]; this is because the angiogenic actions of Flt-1 are not as powerful as those of Flk-1. When VEGF binds to Flt-1, there is less VEGF available to bind Flk-1. Similarly, sFlt-1 acts as an antagonist for VEGF, sequestering it in the plasma [40]. Although these anti-angiogenic effects may seem harmful, they are essential to prevent overexposure to VEGF and maintain homeostasis. For example, sFlt-1 has been shown to be an important factor in the cornea of the eyes; it aids in preserving the corneal avascularity [40]. Under normal physiological conditions, the balance between pro- and anti-angiogenic factors maintain the homeostasis of the vascular system, but as will be described later, when this balance is disturbed, serious pathological consequences can occur.

#### Molecular biology of sFlt-1

Although sFlt-1 is often considered single factor, there are at least four splice variants in humans [41]. All four of these variants contain the first six of seven N-terminal immunoglobulin-like motifs of the receptor, which comprises the extracellular portion of the protein [17]. Given that the first six domains are preserved, normal binding of the receptor’s ligands, VEGF and PlGF, can occur, because it is the second and third motifs that are essential for ligand binding [42, 43]. Whereas the first six motifs are identical among the different sFlt-1 variants, the C-terminus of the isoforms is unique to each one [17]. The isoforms of sFlt-1 produced are both species- and cell-specific. The regulation of the splice variants is believed to take place at both the transcriptional and translational level, but the mechanisms behind regulation are not well understood [41]. One condition which has been shown to upregulate sFlt-1 expression is hypoxia; this likely advances the pathogenesis of preeclampsia [44]. The alternative splicing of sFlt-1 is regulated at the level of mRNA. Both sFlt-1 and Flt-1 are transcribed from the FLT1 gene and have the same start site as well as transcriptional regulatory sequences [45]. This splicing regulation has been shown to be acted on by factors such as VEGF. With increased VEGF exposure, sFlt-1 expression has been shown to be upregulated [46]. Increased expression of VEGF in hypoxic conditions could potentially be a mechanism which promotes sFlt-1 upregulation in preeclampsia.

### sFlt-1 in preeclampsia

#### sFlt-1 imbalance in preeclampsia

As discussed above, under-perfusion of the placenta leads to ischemia and hypoxia of the fetal-placental unit [47]. Studies performed by Fan et al. examined the decidual-placental interface of normal pregnant and preeclamptic women. Using in situ hybridization to determine expression of VEGF and sFlt-1, the researchers observed a significant increase in VEGF in the decidua of preeclamptic women. The authors suggested this as a possible mechanism for the significant increase of sFlt-1 in placental trophoblasts observed in these women [48]. Though the hypoxic placenta secretes increased levels of VEGF [49], the expression of sFlt-1 increases to a greater extent. The imbalance in the pro- and anti-angiogenic factors produced and released into the maternal circulation is believed to drive the maternal syndrome [50]. The imbalance can be seen as an increase in plasma sFlt-1 levels and a decrease in both free VEGF and free PlGF [51]. After delivery, sFlt-1 levels return to normal and the maternal syndrome is alleviated [51], demonstrating the important role of this protein in the pathogenesis of preeclampsia.

#### Potential marker for early preeclampsia diagnosis

Although the exact etiology of preeclampsia remains unclear, there is an ongoing search for biomarkers that could be used to predict the development of preeclampsia in susceptible women. Preeclampsia is associated with an increase in plasma sFlt-1 and a decrease in both plasma VEGF and PlGF [17]. Whereas sFlt-1, with its molecular weight of 110 kDa, is too large to be filtered by the glomerulus; both PlGF and VEGF (30 and 45 kDa respectively) are easily filtered by the kidneys and excreted in the urine. However, in addition to the VEGF made by the vascular endothelium and other cell types, podocytes surrounding the glomerulus also produce VEGF [52]. Therefore, VEGF measured in the urine may not necessarily represent circulating levels accurately, but PlGF, which can only be found in the circulating blood, is more likely to give an accurate representation of the angiogenic state [53]. For this reason, researchers have studied heavily urinary levels of PlGF as a predictive marker for preeclampsia.

Preeclamptic and normotensive women were studied before and after 32 weeks of gestation to compare their urinary PlGF levels. Throughout the study, the preeclamptic women exhibited significantly lower urinary PlGF levels (18 ± 11.3 vs 205 ± 132 pg/mL; *p* < 0.0001); this level of statistical significance was maintained after the PlGF was normalized to urinary creatinine levels. Interestingly, three women who were initially normotensive but went on to develop preeclampsia exhibited lower levels of urinary PlGF, similar to those of the preeclamptic group. Though the researchers suggested limitations to the study, such as small population size, and the need for non-preeclamptic hypertensive pregnancy patients, these data suggest that PlGF has the potential to be an important marker to predict the development of preeclampsia [53].

Furthermore, the ratio of plasma sFlt-1/PlGF has shown to be very effective in predicting the development of preeclampsia in high risk women. Kim et al. [54] found significantly elevated levels of sFlt-1 and significantly decreased levels of free PlGF at 14 to 23 weeks of gestation in women who went on to develop preeclampsia. These findings were observed even prior to the development of hypertension. It was found that a plasma log[sFlt-1/PlGF] ratio greater than 1.4 was associated with an increased risk of developing preeclampsia; this cut-off exhibited an 84% sensitivity and a 78% specificity [54]. Although measurement of urinary PlGF is non- invasive, the plasma ratio of sFlt-1/PlGF may be a more sensitive predictive measure for the development of preeclampsia.

#### sFlt-1 administration leads to preeclamptic phenotype

Several groups have developed experimental models of preeclampsia which highlight the importance of sFlt-1 in the pathogenesis of the disease. Bridges and colleagues [55] were the first to demonstrate that parenteral administration of sFlt-1 by osmotic pump infusion in pregnant rats lead to a preeclamptic-like phenotype. Pumps containing recombinant sFlt-1 or vehicle are inserted on gestation day 13. The pumps will continue to infuse their solution for the remainder of the pregnancy. These animals are later observed to display hypertension, proteinuria, and angiogenic imbalance, having decreased levels of circulating VEGF with the introduction of sFlt-1 [55]. The sFlt-1 model has also been used to demonstrate the direct link between sFlt-1 and vasoconstriction by endothelin-1 through the ET_A_ receptor [56].

Additionally, Maynard et al. [51] infected pregnant rats with an adenovirus containing sFlt-1 or a simple reporter gene on the vector. This was done on gestation day 8 or 9 via injection into the tail vein. Blood pressure measurements were taken on gestation day 16 or 17, and significant blood pressure elevation was observed in animals overexpressing sFlt-1. Free VEGF and PlGF were also significantly decreased in sFlt-1 overexpression. Serum from these preeclamptic and normal rats was additionally used to assess tube formation in endothelial cells, and serum from the preeclamptic sFlt-1 overexpressing animals lead to a significant reduction in tube formation [51]. Further studies using this model of sFlt-1 overexpression have gone on to show a significant decrease in placenta and pup weight in mice overexpressing sFlt-1, along with an increased endothelial dysfunction and increased circulating white blood cells, suggesting immune system activation [57, 58]. Many models of preeclampsia also display an increase in sFlt-1 without outside introduction of the protein, as shown later in this review. This fact, along with the findings that sFlt-1 leads to a preeclamptic phenotype, demonstrates the prominent role of sFlt-1 in the pathogenesis of preeclampsia.

#### sFlt-1 removal from preeclamptic women leads to better outcomes

Apheresis is a therapy which can be used to remove substances from the blood. This has been shown to be safe in men and women for the removal of excess lipids in familial hypercholesterolemia due to the column’s negative charge and the circulating factors’ positive charge [59]. Apheresis has additionally been shown to be safe in pregnant women suffering from familial hypercholesterolemia [60]. This therapy may also be used to remove excess sFlt-1 from the maternal circulation, while retaining the placental sFlt-1, which may be important for maintaining the angiogenic balance in the developing placenta [48]. In a study by Thadhani et al. [61], 11 women with preeclampsia underwent apheresis therapy to remove sFlt-1 from their circulation. There was a mean reduction of plasma sFlt-1 of 18%, which was observed along with a 44% reduction in total protein/creatinine ratio. Women with preeclampsia who did not receive treatment delivered an average of only 3 days after the time of admission, while women who received one treatment had their pregnancies extended an average of 8 days after time of admission, and women receiving multiple treatments had their pregnancies extended 15 days after admission. No adverse effects from the apheresis were reported upon examination of mother and fetus [61].

There are two important caveats to this study. First, it was a small cohort of patients, and larger trials are still needed. Second, the apheresis cartridges used in this study removed sFlt-1 by simple ion-exchange chromatography and were not specific for sFlt-1 itself. It is possible therefore, that additional factors removed by the procedure could have been partially responsible for the beneficial effects noted. Despite these limitations, this study was the first to suggest that in human patients, targeting sFlt-1 in the maternal circulation can potentially relieve some of the preeclamptic symptoms.

## VEGF therapy in preeclampsia

### Benefits of VEGF therapy in preeclampsia

Because angiogenic imbalance has been demonstrated to be of great importance, using VEGF as a therapeutic for preeclampsia to correct the angiogenic imbalance has been observed in several preeclamptic models. VEGF activation of eNOS, with subsequent production of nitric oxide, leads to vasodilation. Nω-nitro-l-arginine methyl ester (L-NAME) is a nitric oxide synthase inhibitor and thus prevents VEGF-induced vasodilation by halting the production of NO [62]. Wistar rats treated with L-NAME have been used as a model of preeclampsia because the animals exhibit many signs of preeclampsia including hypertension and proteinuria. VEGF was given as both a treatment after hypertension and proteinuria were observed as well as a prophylactic when L-NAME treatment was initiated. While the administration of L-NAME alone caused a significant increase in blood pressure, there was no difference in blood pressure between the control group and the prophylactically treated VEGF group (*p* > 0.05), and the group treated after the onset of hypertension had their hypertension almost abolished after administration of VEGF (*p* < .05). Additionally, in this model of preeclampsia, lower platelet count, decreased pup, and placental weight were observed, all of which were reversed by the VEGF treatment [63].

Li and colleagues [64] used the sFlt-1 overexpression model of preeclampsia, described above, to study the effects of VEGF administration as a potential therapeutic for preeclampsia. Pregnant Sprague Dawley rats were injected with an adenovirus which either contained only a reporter gene (control animals) or the sFlt-1 gene (preeclamptic animals). Recombinant human VEGF121 (rhVEGF121) or vehicle control was then given subcutaneously for 6 days. Preeclamptic animals that received the rhVEGF121 had a significant reduction in blood pressure compared to their vehicle-treated counterparts. The treated animals also had a non-significant trend to decrease in urinary protein. Recombinant VEGF121 treatment had little to no effect on control animals. The authors noted the impossibility of extending the effects of rhVEGF121 to humans due to human pregnancies being exposed to elevated sFlt-1 levels for months compared to the short gestation of only 3 weeks in rats. Human pregnancies also have a number of compounding factors that cannot be easily mimicked in a laboratory setting [64].

The reduced uterine perfusion pressure (RUPP) rodent model of surgically induced placental ischemia [65] has also been used to test the efficacy of rhVEGF121 administration. Whereas in humans, the mechanism behind the spontaneous reduction in blood flow to the placenta remains unclear; the RUPP procedure, performed on gestation day 14, involves a mechanical disruption of blood flow. Briefly, silver clips are placed around the aorta above the bifurcation of the iliac arteries and on both ovarian arties. The ovarian clips are important to prevent compensatory increases in blood flow to the uterus. This physical obstruction by the clips leads to an approximate 40% reduction in blood flow to the placentae and developing pups [66]. RUPP animals exhibit many of the symptoms seen in women with preeclampsia including hypertension, proteinuria, reduced GFR (glomerular filtration rate) [67], and increased fetal demise and intrauterine growth restriction (IUGR) [68]. RUPP animals have also been shown to have increased levels of plasma sFlt-1 associated with a decrease in both free VEGF and PlGF compared to normal pregnant counterparts [69], demonstrating the importance of sFlt-1 in the pathophysiology of this model. To test the hypothesis that recombinant VEGF therapy will attenuate the hypertension in the RUPP model, rhVEGF121 therapy was administered via osmotic mini pump on gestational day 14 when the RUPP and sham surgeries were performed. Mean arterial pressure Mean arterial pressure (MAP), as measured by carotid catheter, increased significantly in the RUPP versus normal pregnant animals, and a significant decrease in MAP was observed in the RUPP animals given VEGF therapy versus those receiving vehicle. Additionally, the animals receiving VEGF therapy had their GFR restored to normal [70].

The BPH/5 mouse is a potential model of a spontaneous preeclampsia [71]. Briefly, this mouse was created by inbred brother-sister mating of the hypertensive BPH/2 mouse, creating the “borderline hypertensive” strain. BPH/5 mice have a slightly higher blood pressure at baseline compared to C57 mice, which are used as control. When these animals become pregnant, blood pressure remains stable through the second trimester. Upon entering the third trimester, however, blood pressure of the BPH/5 mice becomes significantly elevated. Urinary protein measured before pregnancy, mid-gestation, and late gestation also shows that the BPH/5 exhibits significantly higher urinary protein in late gestation. Additionally, BPH/5 litters have significantly fewer live births, and the pups have significantly lower birth weights [71]. Free plasma VEGF has also been shown to be significantly decreased in BPH/5 mice compared to C57 controls. For this reason, reintroduction of VEGF (or a LacZ control reporter gene) via adenovirus on gestation day 7 was studied to test the ability of VEGF to rescue the BPH/5 pregnant mouse from its preeclamptic phenotype. BPH/5 mice receiving the VEGF-adenovirus maintained a significantly reduced blood pressure compared to BPH/5 receiving the LacZ-adenovirus control vector. The VEGF treatment had no effect on the blood pressure of the C57 mice. Though VEGF treatment was useful in rescuing the blood pressure elevation and proteinuria in pregnant BPH/5 mice, this therapy did nothing to increase the placental or fetal weights [72].

Bergmann et al. [73] studied the effects of VEGF therapy in preeclampsia using a mouse model of sFlt-1 and VEGF co-administration by adenovirus. An adenovirus containing GFP was used as a control. The pregnant mice given sFlt-1 only exhibited a significantly higher systolic and diastolic blood pressure. Co-administration of sFlt-1 and VEGF had an initial drop in blood pressure below normal levels, which returned to normal levels after 2 days. Additionally, the urinary albumin/creatinine ratio of the co-treated animals was similar to that of the controls (animals given only a vector containing GFP), which was significantly lower than mice receiving only the sFlt-1 adenovirus [73].

### Safety considerations for VEGF as a therapeutic

While the studies above demonstrated the efficacy of VEGF as a potential therapeutic option for preeclampsia, the safety of this growth factor was not demonstrated. It has been shown that mice which overexpress VEGF die in utero between embryonic days 12.5 and 14.5 from cardiac failure. Though they appear to have normal vessel development through embryonic day 11.5, after this, they show signs of edema, characteristic of congestive heart failure. Additionally, the blood vessels have increased permeability, and the ventricular wall of the hearts is abnormally thin in these animals compared to their control littermates. The coronary vessels of the VEGF-overexpressing mice were much larger than normal, and many of them did not connect to another vessel, or were “blind-ended.” This observation suggests that the vessels, under the influence of VEGF, originated from angiogenesis and enlarged without connecting to other vessels. The interventricular septum of the heart in these embryos also exhibited a substantial enlargement to the trabecular layer. The *lacZ* reporter gene from the overexpression vector was found at high concentrations in the trabecular layer of the pup hearts, suggesting that VEGF is of great importance in the development of the heart, and the overexpression of VEGF lead to the abnormalities observed in these animals [74]. Though it is unlikely that VEGF is able to cross the placenta, given these hazardous effects of increased VEGF exposure to fetal development, it is crucial to consider the safety of VEGF as a therapeutic in pregnant women.

Fan et al. [48] also performed a study which involved infecting mice with an endometrium-specific VEGF overexpression viral vector prior to pregnancy, such that, when the mice became pregnant, only the placenta would overexpress VEGF. This proved to be extremely detrimental to the pregnant mice who received this vector compared to the controls, expressing only a reporter gene. The VEGF overexpressing mice had an increased number of reabsorption sites and a decreased number of viable pups at the end of gestation. Fetuses of VEGF overexpressing animals which survived had lower weights and smaller placentas. Whereas the expression of Flt-1 and Flk-1 did not change, sFlt-1 expression levels significantly increased in the mice overexpressing VEGF in their placentas. This suggests that increased VEGF exposure over a long period of time can upregulate sFlt-1 expression and lead to detrimental outcomes in the pregnancy [48]. Because sFlt-1 is one of the prominent drivers of preeclampsia, anything that will increase its expression may lead to a vicious cycle in driving the maternal syndrome. Though poor outcomes were not observed in the VEGF therapy studies mentioned above, these studies did not measure sFlt-1 expression as a consequence of the VEGF therapy and instead measured only free sFlt-1 levels in the circulation. The short gestation period of the animals could also prove too short a time to see significant findings in the developing embryos.

A recent study by our group [75] utilized the drug delivery system elastin like peptide (ELP) fused to VEGF. This ELP-VEGF construct allows the VEGF to bind its receptors normally and exert its actions [76], and importantly, ELP protects VEGF from rapid degradation, extending its plasma half-life. ELP is a synthetic biopolymer which is based on the human elastin gene [77], making it nonimmunogenic [78]. The polymers also aggregate at high temperatures, a process which reverses when temperature is lowered again, making purification of ELP by centrifugation above and below its transition temperature a simple task [79]. It has also been shown that ELP does not cross the placenta, making it an especially ideal candidate for a drug delivery system during pregnancy [80]. The ELP-VEGF construct was given to pregnant rats that underwent either the RUPP surgery mentioned above or a sham operation. At the same time, an osmotic pump was inserted containing the ELP-VEGF therapy, and two doses were examined throughout the study. Though the animals that received the RUPP surgery and received ELP-VEGF therapy had a reduction in blood pressure and decreased free plasma sFlt-1 compared to RUPP animals given saline, there were also negative consequences observed. Total sFlt-1 protein was increased in all animals given the ELP-VEGF construct, though when free sFlt-1 was assessed by ELISA, it was dramatically reduced in the ELP-VEGF treated animals. This suggests that ELP-VEGF induces sFlt-1 production, but it also binds and sequesters the excess sFlt-1, effectively restoring angiogenic balance. Additionally, animals given the higher dose of 10 mg/kg versus 5 mg/kg developed ascites and tissue encapsulation around the osmotic pump. It is likely that the ascites was the result of increased vascular permeability induced by intraperitoneal administration of ELP-VEGF, and the therapeutic window of the novel drug is potentially narrow [75]. Again, given the short gestation period of these animals, it may be difficult to observe the long-term effects of ELP-VEGF treatment, but it may be possible to optimize the dose and/or delivery route to minimize the acute side effects.

Correction of the angiogenic imbalance in preeclampsia is crucial. Delivery of VEGF as a therapeutic has been shown to attenuate the hypertension in several models of preeclampsia, and it is possible to restore this angiogenic imbalance. However, there remains the risk of VEGF toxicity if the dosage is not correct. Though it is unknown if VEGF will cross the placenta, especially due to VEGF’s actions to increase vascular permeability, the development of the fetal heart can be greatly impaired if overexposure were to occur, and the evidence suggests there may be serious dose limiting side effects in the mother. An agent which can correct the angiogenic imbalance while not activating the Flk-1 receptor would be a more favorable option to avoid these off-target effects.

## Placental growth factor

### Expression

Placental growth factor (PlGF) has also been studied as a potential therapeutic option to correct the angiogenic imbalance that occurs during preeclampsia. Despite the fact that it shares only 42% amino acid identity with VEGF-A, it, too, is a member of the VEGF family [81]. Though the name refers to the placenta [82], PlGF is actually expressed at low levels by several other organs including the heart, thyroid, and lung [83, 84]. This protein has four different isoforms, which are produced as a result of alternate splicing. The main difference between the isoforms is their ability to bind heparin (PlGF-2 and PlGF-4) or their inability to bind heparin (PlGF-1 and PlGF-3) [85]. This growth factor is glycosylated and circulates as a homodimer. Glycosylation is important for receptor binding, as when there is a mutation leading to decreased glycosylation, there is a decreased affinity for receptor binding [81].

### PlGF and angiogenesis

The physiological function of PlGF has not been well understood. Whereas VEGF is responsible for the majority of angiogenesis and endothelial cell health, PlGF has been shown to be a very weak stimulator of endothelial cell proliferation and signaling [85]. By itself, PlGF has relatively no impact on angiogenesis or endothelial cell proliferation. However, as mentioned previously, binding of PlGF to Flt-1 can displace VEGF, freeing it to bind to the lower affinity Flk-1 receptor. This is particularly important when VEGF bioavailability is low [86]. Additionally, PlGF has been shown to be extremely important in pathological states. When comparing *Plgf*^−/−^, *eNos*^−/−^, and *Plgf*^−/-^*eNos*^−/−^ mutant mice, the mice which were deficient in both PlGF and eNOS exhibited increased hind limb ischemia, decreased capillary density, and a 47% increase in death rates. Whereas the *eNos*^−/−^ mice were also shown to have ischemia and decreased capillary density, the disruption of both PlGF and eNOS simultaneously exacerbated these effects. Due to the ischemia, VEGF was measured and found to be upregulated. Because the capillary density remained low even with increased production of VEGF, the value of PlGF in pathogenic conditions to promote angiogenesis is demonstrated [87].

VEGF and Flk-1 are expressed to a greater extent during the earlier months of pregnancy, but their expression levels decline during the last few months of gestation [88]. PlGF and Flt-1, on the other hand, have increased expression as the pregnancy progresses closer to term [89]. These expression patterns, as well as the corresponding placental villous vascular growth, suggest that VEGF and Flk-1 are essential during the first and second trimesters of pregnancy to create a heavily branched network of capillary beds of the mesenchymal and intermediate villi. During the final trimester, though, PlGF and Flt-1 are engaged in the formation of long, terminal capillary loops with little branching [90]. This, along with the diagnostic value of sFlt-1/PlGF in predicting preeclampsia, demonstrates the importance of PlGF expression in late pregnancy to prolong gestation.

### PlGF as a therapeutic for preeclampsia

As stated above, PlGF is considered pro-angiogenic when it binds its receptor, Flt-1, because this increases the bioavailability of VEGF. Considering sFlt-1 has the same binding capabilities as Flt-1, PlGF is also able to bind this soluble protein readily. With this knowledge, and the goal of preventing the negative effects of VEGF therapy, several researchers have set out to investigate the benefits of PlGF as a therapeutic option for preeclampsia.

Recombinant human PlGF (rhPlGF) was tested in the reduced uterine perfusion pressure (RUPP) model of preeclampsia in rats. Again, this model induces a preeclamptic-like state by performing surgery on gestation day 14 to place silver clips on abdominal aorta and ovarian arteries to reduce blood flow to the developing pups. When RUPP animals were given rhPlGF, their hypertension was abolished such that it was not statistically different from the control animals. Treatment with rhPlGF also returned GFR to normal levels, with the untreated RUPP animals experiencing lower GFR. The rhPlGF significantly reduced the circulating levels of free sFlt-1 in the RUPP-treated animals compared to the RUPP animals that received vehicle. Additionally, 8-isoprostane, which is a marker of oxidative stress, was found to be significantly higher in the RUPP animals compared to all other groups, suggesting the rhPlGF is able to reduce oxidative stress in this model [91].

In another relevant model of preeclampsia, baboons have been studied to see the effects of rhPlGF therapy using surgically induced uteroplacental ischemia (UPI). Similar to the RUPP model, UPI involves a mechanical disruption of blood flow by ligation of a single uterine artery in the pregnant primate, accounting for an approximate 40% blood flow reduction to the developing offspring. The baboons also exhibit signs of preeclampsia such as hypertension and endothelial dysfunction. Histological changes in the kidneys of these animals have also been found to be identical to those of preeclamptic women. Furthermore, baboons undergoing the UPI procedure have significantly higher levels of circulating sFlt-1 compared to the sham control primates [92]. In order to test rhPlGF therapy, Makris and associates [93] implanted telemeters into the baboons for blood pressure measurements, allowed the animals to recover, and performed the UPI or sham surgery. Within 3 days of inducing UPI, the increase in blood pressure was statistically significant. After treatment with rhPlGF began, 3 days passed before the blood pressure became significantly decreased in the primates compared to those receiving vehicle treatment. The decrease in blood pressure was associated with a decrease in proteinuria, though this was not statistically significant until day five of treatment. Interestingly, mRNA of sFlt-1 was also measured from the placentas of these animals after delivery, and a significant decrease in sFlt-1 expression after rhPlGF treatment compared to vehicle was observed, which experienced a slight increase in sFlt-1 expression [93]. These data showing the efficacy of PlGF, as well as its effect to antagonize sFlt-1, support its potential to be a viable therapeutic option for the treatment of preeclampsia. One drawback of PlGF as a therapy is its relatively short half-life [94], limiting the route of administration. Our lab group has recently begun experimenting with a novel peptide-based drug delivery system for use in pregnancy which excludes fetal exposure [76]. We have recently constructed a PlGF-based chimeric protein with improved pharmacokinetic parameters, and efficacy of the peptide is currently being assessed.

## Conclusions

Preeclampsia is a dangerous multifactorial disease which presents during the second half of pregnancy and results in maternal hypertension among other symptoms. An angiogenic imbalance, resulting from an inappropriately elevated increase in sFlt-1 expression, has been shown to drive the progression of the maternal syndrome. To date, the only effective treatment of preeclampsia is delivery of the fetus and placenta. Targeting sFlt-1 seems an intuitive method for new therapeutic approaches. While this could theoretically be accomplished by RNAi technology, the multiple splice variants and high sequence homology of sFlt-1 to the full length receptor makes this problematic. Apheresis techniques, while showing early promise in managing the disorder, are likely not cost or labor-effective in any but the most severe cases of preeclampsia. Therefore, the use of VEGF family-based therapeutics to target sFlt-1 appears to be the most viable option from a practical standpoint for the majority of cases. Further, while both VEGF and PlGF supplementation have shown to be effective in relieving the hypertension and many of the accompanying symptoms such as proteinuria, decreased pup weight, and low platelet count, VEGF may also exhibit dose dependent toxicity. Not only does VEGF have negative effects on developing embryos when they are exposed to high doses, but pregnant rats who received large doses of VEGF showed signs of edema formation. Because PlGF does not bind the more active angiogenic receptor, Flk-1, these negative side effects are not observed when treatment is administered. This suggests that pro-angiogenic therapy is a viable option for the treatment of preeclampsia, but VEGF may have a narrower therapeutic window, making PlGF a safer option. Based on the available clinical and preclinical data, we believe that modified, stabilized members of the VEGF family, like PlGF, could finally provide a potential therapeutic for this currently confounding and devastating disorder.

## Abbreviations

ELP: Elastin-like polypeptide
eNOS: Endothelial nitric oxide synthase
Flk-1: Fetal liver kinase-1
Flt-1: Fms-like tyrosine kinase-1
GFR: Glomerular filtration rate
IUGR: Intrauterine growth restriction
L-NAME: Nω-nitro-l-arginine methyl ester
NO: Nitric oxide
PlGF: Placental growth factor
rhPlGF: Recombinant human placental growth factor
rhVEGF: Recombinant human vascular endothelial growth factor
RUPP: Reduced uterine perfusion pressure
sFlt-1: Soluble fms-like tyrosine kinase-1
UPI: Uteroplacental ischemia
VEGF: Vascular endothelial growth factor
VEGFR-1: Vascular endothelial growth factor receptor-1
VEGFR-2: Vascular endothelial growth factor receptor-2
